# Functional Impact of 14 Single Nucleotide Polymorphisms Causing Missense Mutations of Human α7 Nicotinic Receptor

**DOI:** 10.1371/journal.pone.0137588

**Published:** 2015-09-04

**Authors:** Qinhui Zhang, Yingjie Du, Jianliang Zhang, Xiaojun Xu, Fenqin Xue, Cong Guo, Yao Huang, Ronald J. Lukas, Yongchang Chang

**Affiliations:** 1 Department of Zoology, College of Life Sciences, Sichuan University, Chengdu, Sichuan Province, 610064, China; 2 Chengdu institute of Biology, Chinese Academy of Sciences, Chengdu, Sichuan 610041, China; 3 Division of Neurobiology, Barrow Neurological Institute, St. Joseph’s Hospital and Medical Center, Phoenix, Arizona 85013, United States of America; 4 University of California Los Angeles, Henry Samueli School of Engineering and Applied Science, Los Angeles, California 90095, United States of America; 5 Department of Neurobiology, Beijing Institute of Brain Disorders, Capital Medical University, Key Laboratory for Neurodegenerative Disease of the Ministry of Education, Beijing Center of Neural Regeneration and Repair, Beijing Key Laboratory of Brain Major Disorders; State Key Lab Incubation Base, Beijing Neuroscience Disciplines, Beijing 100069, China; 6 Core Facilities for Electrophysiology, Core Facilities Center, Capital Medical University, Beijing 100069, China; 7 Department of Obstetrics and Gynecology, St. Joseph’s Hospital and Medical Center, Phoenix, Arizona 85013, United States of America; Neuroscience Campus Amsterdam, VU University, NETHERLANDS

## Abstract

The α7nicotinic receptor (nAChR) is a major subtype of the nAChRs in the central nervous system, and the receptor plays an important role in brain function. In the dbSNP database, there are 55 single nucleotide polymorphisms (SNPs) that cause missense mutations of the human α7nAChR in the coding region. In this study, we tested the impact of 14 SNPs that cause missense mutations in the agonist binding site or the coupling region between binding site and channel gate on the receptor function. The wild type or mutant receptors were expressed or co-expressed in *Xenopus* oocytes, and the agonist-induced currents were tested using two-electrode voltage clamp. Our results demonstrated that 6 mutants were nonfunctional, 4 mutants had reduced current expression, and 1 mutants altered ACh and nicotine efficacy in the opposite direction, and one additional mutant had slightly reduced agonist sensitivity. Interestingly, the function of most of these nonfunctional mutants could be rescued by α7nAChR positive allosteric modulator PNU-120596 and agonist-PAM 4BP-TQS. Finally, when coexpressed with the wild type, the nonfunctional mutants could also influence the receptor function. These changes of the receptor properties by the mutations could potentially have an impact on the physiological function of the α7nAChR-mediated cholinergic synaptic transmission and anti-inflammatory effects in the human SNP carriers. Rescuing the nonfunctional mutants could provide a novel way to treat the related disorders.

## Introduction

Cholinergic transmission plays an important role in brain function, such as learning and memory through the neurotransmitter acetylcholine and its receptors [1]. nAChRs are acetylcholine-operated ion channels. They belong to the pentameric ligand-gated ion channel superfamily, which includes vertebrate cation-selective nicotinic receptors [2], serotonin receptor type 3 [3] and zinc-activated ion channel [4], and anion-selective GABA_A/C_ receptors [5–7] and glycine receptors [8]. The pentameric ligand-gated ion channels are allosteric proteins [9], in which, the orthosteric ligand binding sites are located in the extracellular N-terminal domain, and the ion conducting channel is formed by the transmembrane domain. The binding site is formed by 6 binding loops. Loops A, B, C from one subunit forms the principal side of the binding pocket, whereas loops D, E, F from the neighboring subunit form the complementary side of the binding pocket [10]. Thus, neurotransmitter binding to the N-terminal binding site can allosterically (remotely) control the channel gate through an evolutionarily interconnected allosteric network [11, 12]. There are 17 subunits and isoforms of nAChRs: α1–10, β1–4, γ, δ, and ε [1, 2, 13]. The pentameric receptor can have five identical (such as in α7 nAChR) or different (such as in α4β2 nAChR) subunits in a receptor. In the mammalian brain, the major subtypes are heteromeric α4β2 and homomeric α7 nAChRs [2].

Homomeric α7 nAChR is widely distributed in the CNS and autonomic ganglia. It has much higher calcium permeability than other subtypes of nAChRs [1]. In addition to excitatory synaptic transmission, α7 nAChR also plays an important role in transmitter release [14], neurite growth [15], neuronal survival and apoptosis[16] and neuronal plasticity [17]. Dysfunction of α7 nAChR is associated several neurological and psychiatric disorders, such as schizophrenia [18]. In addition, α7 nAChR gene deletion is associated with seizures and mental retardation [19, 20].

Transcript of α7 nAChR subunit is also found in immune cells, especially in macrophages in peripheral blood and microglia cells in the CNS [21]. In fact, nicotine has anti-inflammatory effects, which can be blocked by α7 selective antagonist [22]. Experimental evidence suggests that there are cholinergic anti-inflammatory pathways in peripheral and CNS via α7 nAChR expressed on macrophage or microglia [23, 24]. These studies suggest the essential role of α7 nAChR in inhibiting cytokine synthesis by the cholinergic anti-inflammatory pathway. A more recent study suggests that the α7 nAChR mediates the inhibition of T cell function through the regulatory T cells [25].

Single Nucleotide Polymorphisms (SNPs) are single base variations of the DNA sequence among individuals of the same species [26]. In the coding region of exons, a SNP can cause missense mutations, which result in substitution of an amino acid residue at the protein level, which may have impact on protein expression and/or function.

**Fig 1:**
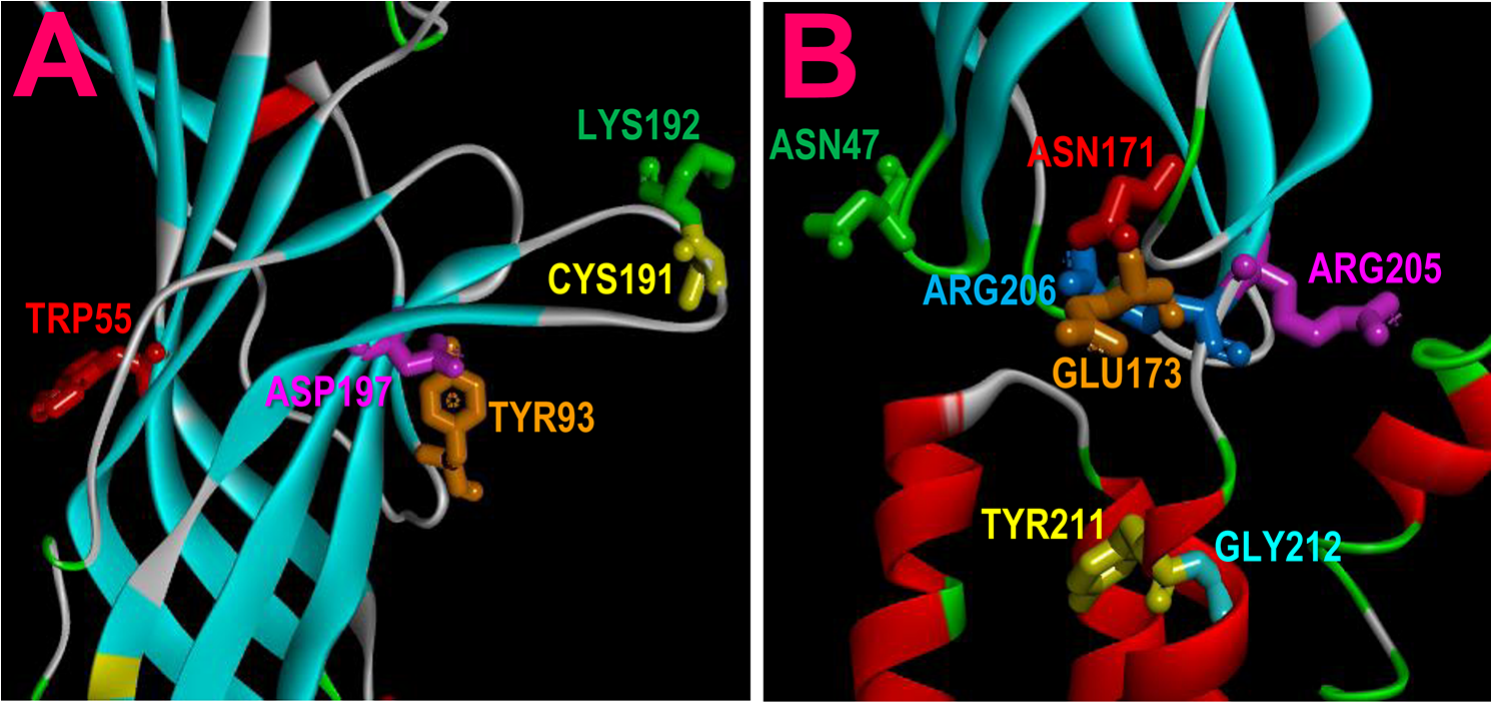
Location of 14 mutations in the α7 nAChR receptor. A: The residues with the mutations in the binding region (loops A (TYR93), and C (CYS191, LYS192, ASP197) of the principal side of a binding pocket, and D (TRP55) of the complementary side of the neighboring binding pocket); B, The residues with the mutations in the coupling region (loop 2 (ASN47), loop 9 (ASN171 and GLU173), pre-M1 (ARG205 and ARG206) and M1 (TYR211 and GLY212).

The SNP database [27], dbSNP, in the National Center for Biotechnology Information (NCBI) listed 55 SNPs causing missense mutations of the human α7nAChR. Mapping these mutations in the 3D structure of the α7 nAChR subunit homology model, we identified 14 α7nAChR SNPs that cause missense mutations in the agonist binding loops or the coupling region between amino terminal domain and transmembrane domain (Fig 1). Specifically, these mutants are located in the binding loop A (Y93C), loop C (C191Y, K192R, and D197N), loop D (W55G); coupling loop 2 (N47D), loop 9 (N171S and E173K), pre-M1 (R205C, R205H, and R206C), and extracellular end of M1 (Y211C, G212V and G212S). In this study, we characterized the functional impact of these 14 mutants for the neurotransmitter acetylcholine, and smoking related agonist nicotine. These SNPs and corresponding mutations are listed in Table 1. It should be pointed out that although in this study we selected the mutants in the binding site and coupling region, we cannot exclude possibility that mutations in other part of the receptor can influence channel function. As demonstrated by single channel studies to map gating energy changes upon mutagenic perturbation in multiple domains, although sources of gating energy are mainly from the binding loops C, B, and A, other part of the receptor can also influence channel gating, but to a lesser extent. [28, 29].

**Table 1:** List of the SNPs of the human α7 nAChR in this study.

SNP name	Missense mutation	Numbering w/o signal peptide	Location in the receptor
**rs201473594**	N69D	N47D	Loop 2 (coupling)
**rs12899798**	W77G	W55G	Loop D (binding)
**rs200908085**	Y115C	Y93C	Loop A (binding)
**rs201108331**	N193S	N171S	Loop 9 (coupling)
**rs201210785**	E195K	E173K	Loop 9 (coupling)
**rs200236230**	C213Y	C191Y	Loop C (binding)
**rs143167432**	K214R	K192R	Loop C (binding)
**rs377100778**	D219N	D197N	Loop C (binding)
**rs140316734**	R227C	R205C	Pre-M1 (coupling)
**rs138222088**	R227H	R205H	Pre-M1 (coupling)
**rs201224804**	R228C	R206C	Pre-M1 (coupling)
**rs142728508**	Y233C	Y211C	M1 (coupling)
**rs201524804**	G234S	G212S	M1 (coupling)
**rs377388459**	G234V	G212V	M1 (coupling)

## Materials and Methods

### Mutagenesis and cRNA Preparation

The cDNA encoding wild type human α7 nAChR subunit was cloned into pGEMHE oocyte expression vector with T7 orientation. The mutations were made with the PCR-based QuikChange method of the site-directed mutagenesis (Stratagene, Hercules, CA, USA) with Phusion DNA polymerase (New England Biolab, Ipswich, MA). The mutations were confirmed by automated DNA sequencing of the entire coding region. The wild type and mutant cDNAs were amplified by PCR with Phusion DNA polymerase and M13 forward and reverse primers, and used as the DNA templates for cRNA synthesis. The cRNAs were transcribed by standard *in vitro* transcription using T7 RNA polymerase. After degradation of the DNA template by RNase-free DNase I, the cRNAs were purified and resuspended in diethyl pyrocarbonate (DEPC)-treated water. cRNA yield and integrity were examined with an Eppendorf BioPhotometer and a 1% agarose gel.

### Oocyte preparation and injection

Oocytes were harvested from female *Xenopus laevis* (Xenopus I, Ann Arbor, MI, USA), using the protocol "Xenopus Care and Use" approved by the Institutional Animal Care and Use Committee of the St. Joseph's Hospital and Medical Center for this study. Briefly, the frog was anesthetized by 0.2% MS-222. The ovarian lobes were surgically removed and placed in the incubation solution consisting of (in mM) 82.5 NaCl, 2.5 KCl, 1 MgCl_2_, 1 CaCl_2_, 1 Na_2_HPO_4_, 0.6 theophylline, 2.5 sodium pyruvate, and 5 HEPES; 50 U/ml penicillin, and 50 μg/ml streptomycin, pH 7.5. The frog was given analgesic xylazine hydrochloride (10mg/kg, ip) and antibiotic gentamicin (ip, 3mg/kg) after surgery, and then allowed to recover from the surgery before being returned to the incubation tank. The animals were euthanized under anesthesia with MS-222 after the third surgery. The ovarian lobes were cut into small pieces and digested with 1 Wunsch unit/ml liberase blendzyme 3 (Roche Applied Science, Indianapolis, IN, USA) with constant stirring at room temperature for 1.5–2 hours. The dispersed oocytes were thoroughly rinsed with the above solution. The stage VI oocytes were selected and incubated at 16°C before injection. Micropipettes for injection were pulled from borosilicate glass on a Sutter P87 horizontal puller, and the tips were cut with forceps to ≈40 μm in diameter. The cRNA was drawn up into the micropipette and injected into oocytes with a Nanoject micro-injection system (Drummond, Broomall, PA, USA) at a total volume of 20~60 nl.

### Two-electrode voltage-clamp

Two to 4 days after injection, the oocytes expressing the wild type or mutant receptors were placed in a custom made small volume chamber with continuous perfusion with the calcium-free oocyte Ringer’s solution, which consisted of (in mM) 92.5 NaCl, 2.5 KCl, 1.8 BaCl2, and 5 HEPES, pH 7.5. The chamber was grounded through an agar KCl bridge. The oocytes were voltage-clamped at-70 mV to measure ACh-, nicotine-, or 4BP-TQS-induced currents using an AxoClamp 900A amplifier (Molecular Devices, Sunnyvale, CA, USA). The current signal was filtered at 50 Hz with the built-in 4 pole low-pass Bessel filter in the AxoClamp 900A and digitized at 100 Hz with Digidata1440A (Molecular Devices).

### Drug Preparation

Acetylcholine chloride and nicotine (SigmaAldrich, St. Louis, MO, USA) PNU-120596 and 4BP-TQS (Tocris, Bristol, UK), atropine (SigmaAldrich) stock solutions were prepared from the solid and stored at-20°C in aliquots. Working concentrations of these drugs were prepared from stock solutions immediately before use.

### Data Analysis

The peak current and net charge of the ACh-induced current were measured using Clampfit10.3. The peak current was measured relative the average baseline within 1 second before ACh application switching. The net charge (the area under the curve) was measured with the start point 0.5 second after the pinch valve switching (~0.5 second before inward current rising) to 4 seconds later, when the fast decay phase of the currents induced by all concentrations of ACh was essentially completed for the wild type and mutants, using the average baseline within 1 second before agonist application switching. The concentration-response relationship of the agonist-induced current or charge in recombinant nAChRs was least-squares fit to a Hill equation with GraphPad Prism 6.0 (GraphPad Software, Inc, La Jolla, CA) to derive the EC_50_ (the agonist concentration required for inducing a half maximal change), Hill coefficient (the slope factor), and maximum current, which was then used to normalize the concentration-response curve from individual oocytes. The average of the normalized currents or charges for each agonist concentration was used to plot the data. All the data were presented as mean ± SEM (standard error). Statistical comparisons for logEC_50_s, agonist-induced currents at a saturation concentration, or allosteric modulator rescued currents between wild type/blank control and multiple mutants were performed with one way ANOVA, or two-sided grouped t-test for two group comparison. Correlation between two parameters was performed by linear regression analysis in Prism6.0.

### Homology Modeling

The homology model of the human α7nAChR subunit was made with ICM Pro 3.7–2C (MolSoft, San Diego, CA) using chain A of the EM structure of *Torpedo* nicotinic receptor (PDBID: 2BG9 chain A) as the template for Fig 1. The resulting models were used to map the SNPs in the 3D structure using Discovery Studio 4.0 Visualizer (Biovia, San Diego, CA). The same software was also used to present the crystal structures of acetylcholine binding protein (AChBP) with ACh or nicotine in the binding pocket (PDBID: ACh binding with AChBP: 3WIP, nicotine binding with AChBP: 1UW6).

## Results

### Influence of mutations on the currents induced by ACh and nicotine

**Fig 2:**
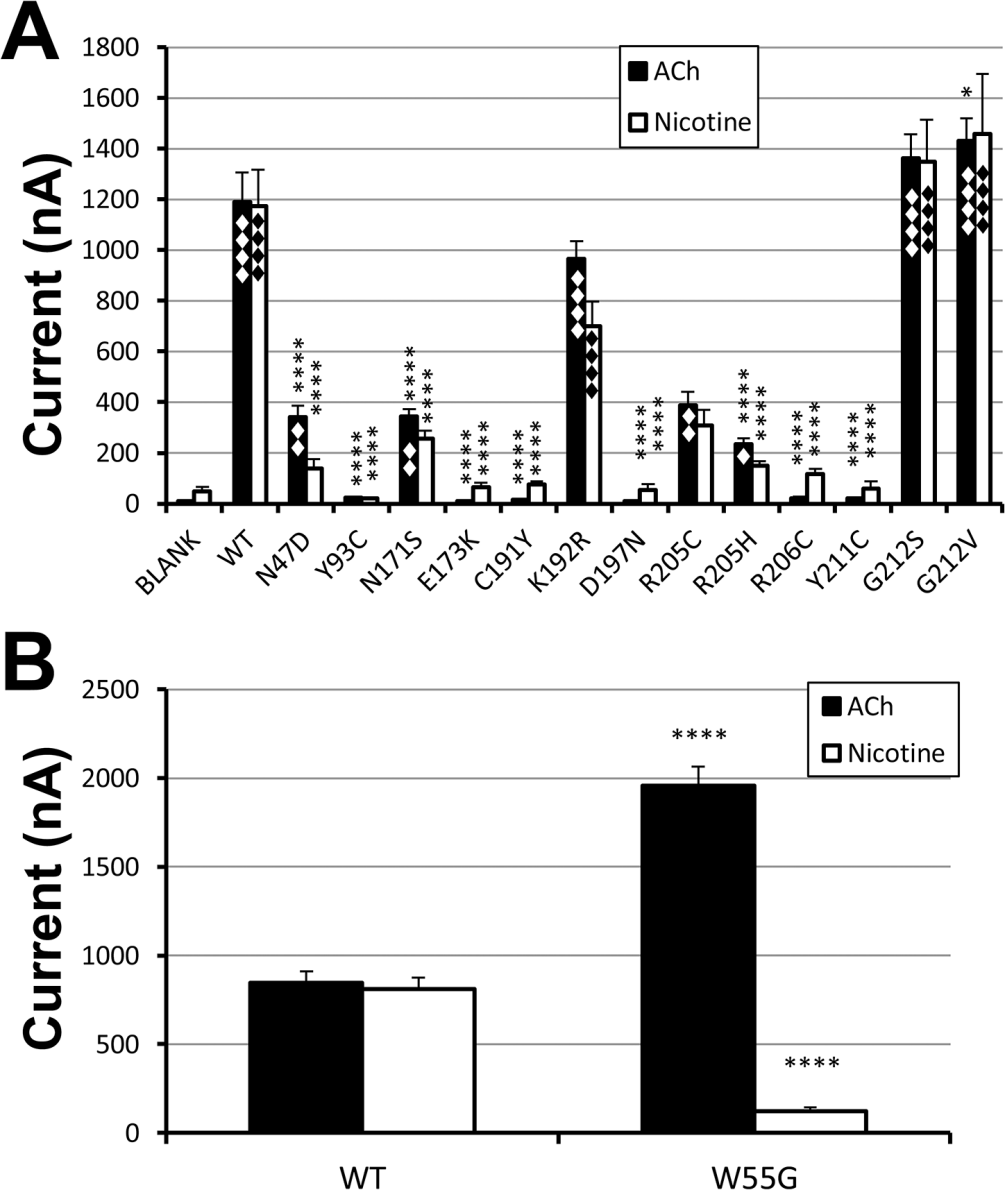
Current responses of the wild type and mutant receptors to 3.16mM ACh or nicotine with non-injected oocyte controls. A. 13 mutants and wild type control along with un-injected oocyte control (for nonfunctional mutants). The same amount of cRNAs for the wild type or mutant receptors was injected. On the 3rd post-injection day, the oocytes were tested with ACh (with 1 μM atropine) or nicotine. The name of each condition is indicated at the bottom of each bar. Each group had 8–18 oocytes from two sets of experiments. Asterisk (*, **, or ****) represents that the difference between the wild type and each mutant is statistically significant (P<0.05, P<0.01, or P<0.0001) in Tukey multiple comparison test of one-way ANOVA. ♦, ♦♦ or ♦♦♦♦ represent the statistical difference with P<0.05, P<0.01, or P<0.0001 between blank and each mutant. B. W55G mutant and its wild type control in a separate experiment (10 oocytes each group). ****: P<0.0001 with 2-sided grouped t-test.

To test the impact of the 13 mutants (excluding W55G, which was tested in a separate experiment, as it was reported in the 2010 Society for Neuroscience Annual Meeting), we injected the same amount of cRNAs of the wild type and 13 mutants into *Xenopus* oocytes and tested receptor function with 3.16 mM ACh, a saturation concentration of the wild type α7 nAChR. Fig 2A shows the average currents induced by ACh for each construct. Note that except for two G212 mutants and K192R, all the remaining mutants exhibited reduced currents when compared to the wild type. Among them, R205C, R205H, N171S, and N47D mutants exhibited 3.5- to 5-fold reduction. But their current levels were still significantly higher than the un-injected control. Interestingly, Y93C, E173K, D197N, C191Y, R206C, and Y211C were nearly insensitive to 3.16 mM ACh. The very low ACh-induced currents are likely due to muscarinic receptor activation by the high ACh concentration, despite the presence of 1μM atropine, because these small currents were not statistically different from the current induced by 3.16 mM ACh in the un-injected oocytes. Thus, these mutants are essentially nonfunctional. The responses to 3.16mM nicotine are also shown in Fig 2A. The current responses to nicotine for all constructs exhibited a pattern similar to that for ACh responses. High concentration nicotine-induced small currents in the same set of six mutants were also not significantly different from that from the blank oocytes. Due to higher background current induced by 3.16mM nicotine in un-injected control and lower nicotine-induced current, the nicotine-induced currents in R205C, R205H, N171S, and N47D mutants did not reach statistical significance when compared to the un-injected control. The mechanism for the nonspecific effect of high concentration nicotine on the oocytes is unknown. However, we have noticed that it is oocyte batch dependent. In summary, out of 13 mutants tested, only three mutants showed similar current levels as the wild type. There were four functional mutants with reduced currents, and six nonfunctional mutants.

For the W55G mutation, our results for ACh responses were similar to a previous report by Williams et al [30]. In addition, we also noticed that W55G mutation differentially altered the ACh and nicotine responses. Fig 2B shows that W55G mutation dramatically increased the 3.16mM ACh-induced current, but dramatically decreased 3.16mM nicotine induced current. We will discuss the potential mechanism for this difference later.

### Concentration-response relationships of functional mutants and current kinetics

**Fig 3:**
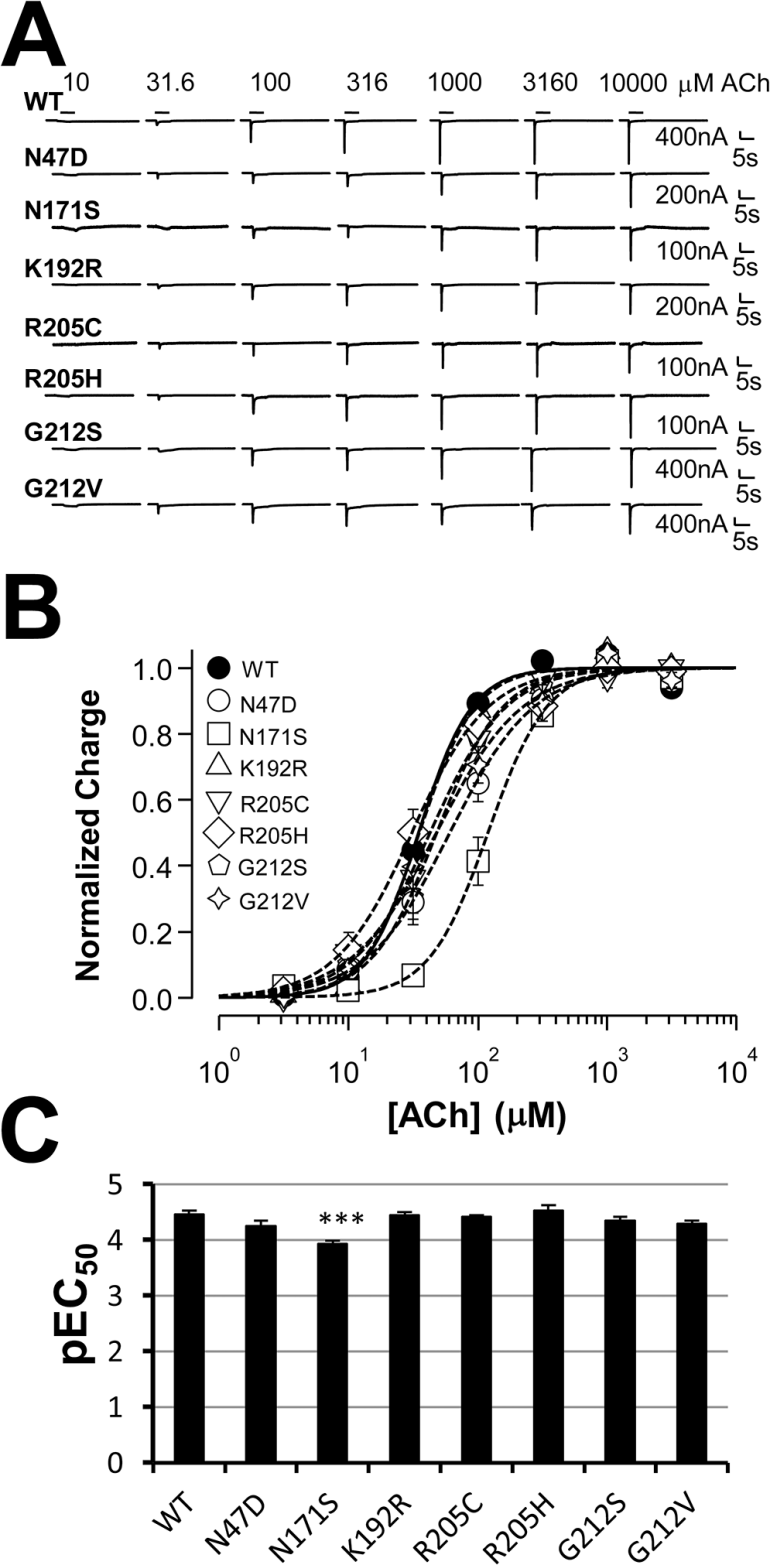
ACh concentration-response for most of the functional mutants. **A**. Representative current traces induced by ACh for the wild type and mutant receptors with the concentrations indicated. **B**. Averaged and normalized concentration-responses of charge (6–8 oocytes each group). Lines are nonlinear least squares fits of the normalized averages of the responses to the Hill equation. **C**. Bar graph of the pEC50 values (negative logEC_50_s) derived from B. ***: P<0.001 when compared to the WT value.

**Fig 4:**
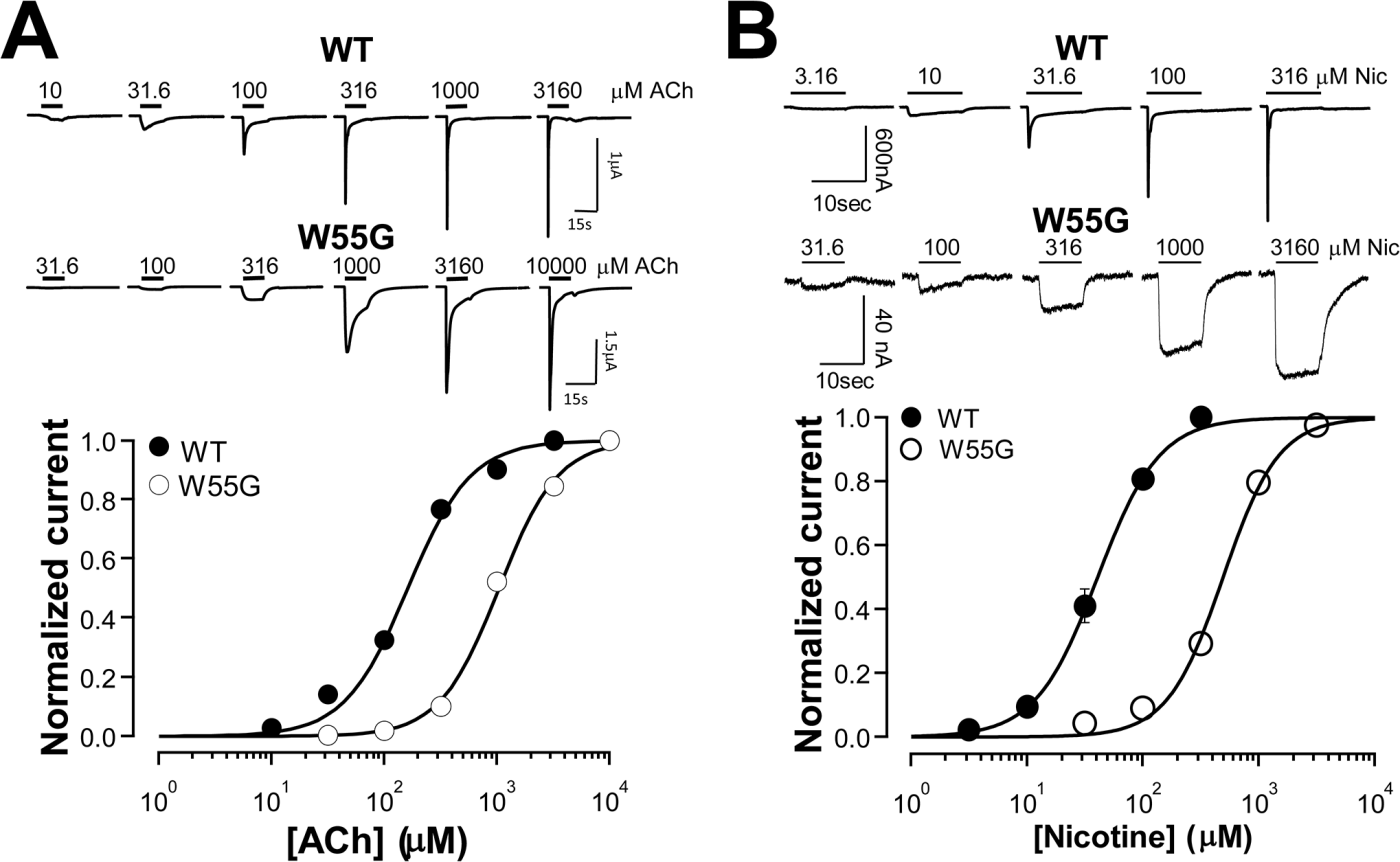
Concentration-responses of W55G mutant to ACh and nicotine. A. Concentration responses of the wild type and W55G to ACh. Top: raw current traces; bottom: normalized and averaged currents. Lines are least-squares fit of the data to the Hill equation. The resulting EC_50_ for ACh in the wild type receptor was 210.5±24.3 μM, and the EC_50_ for W55G mutant was 1375.3±130.5 μM (N = 5). B. Concentration responses of the wild type and W55G to nicotine. Top: raw current traces; bottom: normalized and averaged currents. Lines are least-squares fit of the data to the Hill equation. The resulting EC_50_ values for nicotine were 43.6±4.8 μM and 530.40±12.91 μM for the wild type and mutant receptor respectively (N = 5).

To further characterize sensitivity of the functional mutants, we performed concentration response analysis. Fig 3A and 3B shows that 7 functional mutants were similar to, or only slightly deviated from, the wild type in their ACh sensitivity. Their pEC_50_s (negative log of the concentration required to activated 50% charge) are plotted in Fig 3C. Note that, only N171S mutant had slightly lower sensitivity than the wild type statistically significant with charge analysis. For their current decay kinetics, there was no obvious difference that we can resolve with our recordings. For nicotine concentration-response, because high concentration of nicotine could induce small currents in un-injected oocytes, we could not get clean concentration-response relationships for these mutants (data not shown). In contrast, the W55G mutant exhibited larger sensitivity shift with a 6.5-fold reduction of the receptor sensitivity to its natural agonist, ACh (Fig 4A), and a 12-fold reduction for nicotine sensitivity (Fig 4B). By examining the current traces, it is also noticeable that the decay rate of the ACh-induced current is slower in W55G mutant, similar to a previous finding [30]. In addition, it is obvious that the kinetics of the nicotine-induced current in W55G mutant is much slower than that in the wild type. Fig 4 also demonstrates that 3.16mM nicotine used in Fig 2B is near saturation concentration for both the wild type and W55G mutant, whereas 3.16mM ACh is a saturation concentration for the wild type, but is at about EC80 for W55G. It means that the maximum response of W55G to ACh should be higher than the 3.16mM ACh-induced current. Thus, we can also conclude that the W55G mutation dramatically increased ACh efficacy, but reduced nicotine efficacy. In summary, for the functional mutants, W55G altered the sensitivity to both ACh and nicotine in the same direction and to a similar extent. N171S is the only other functional mutant with a slight decrease in ACh sensitivity.

### Rescuing nonfunctional mutants by positive allosteric modulator

As mentioned above, we have identified 6 mutants, which were essentially non-responsive to 3.16 mM ACh. This non-responsiveness may indicate that the mutant receptors are not functional despite surface expression in the cell membrane. Alternatively, the mutations may influence surface expression or assembly of the receptor. Since these non-responsive mutants are with the mutations in the binding site or coupling region, it is likely that surface expression is normal but function is impaired. These two possibilities will be differentiated by the rescuing effect of positive allosteric modulators or allosteric agonists.

**Fig 5:**
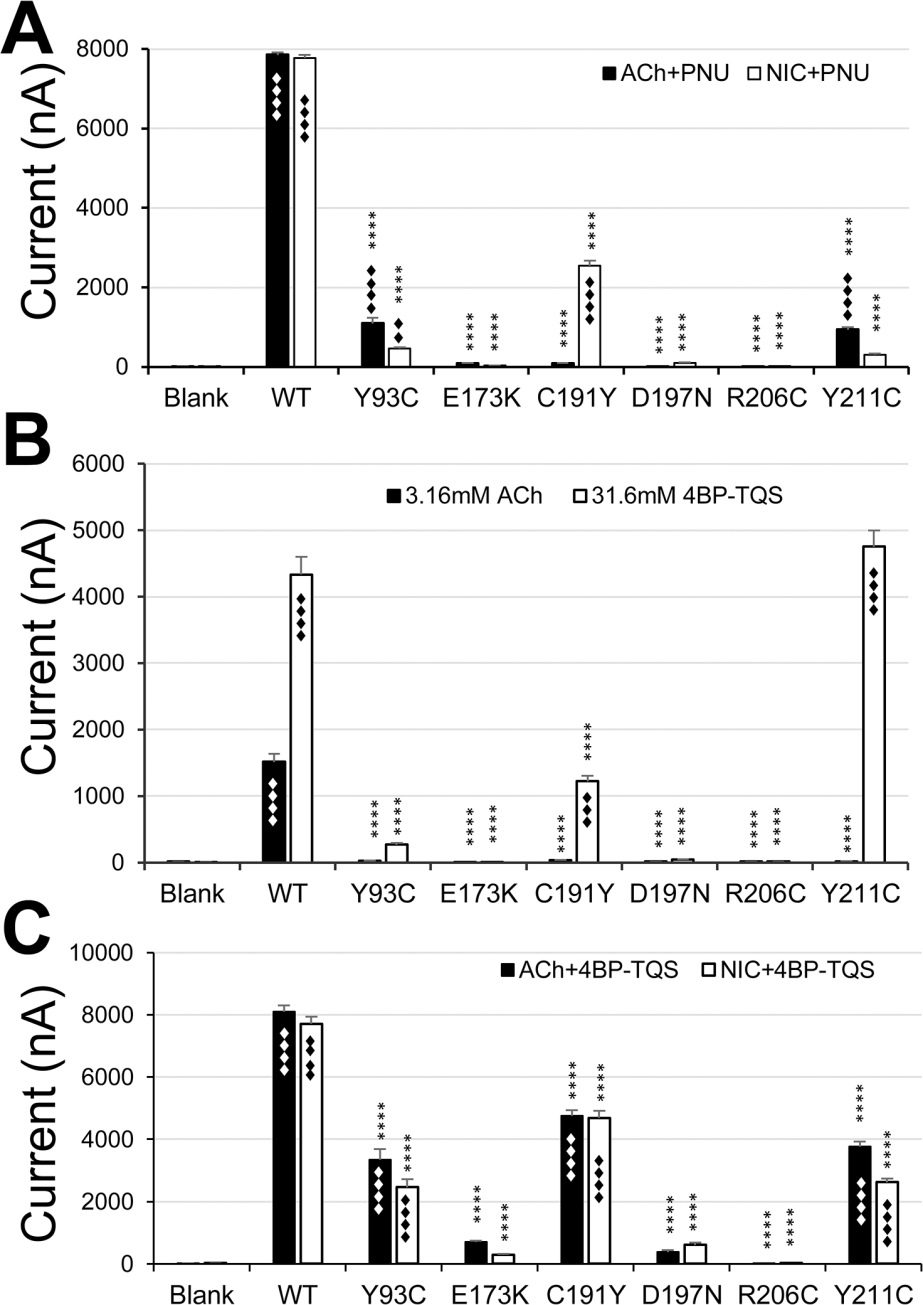
Agonist-responses for the nonfunctional mutants in the presence of a PAM or agonist-PAM. A. Co-application of 31.6 μM PNU-120596 with 200 μM ACh or nicotine rescued the receptor functions for some of the nonfunctional mutants. The same amount of cRNA was injected for each group, and recordings were performed after 3 days in 9–19 oocytes for each group). The bar graph represents the average currents rescued by PNU-120596. In case of the wild type, the current represents the rescued current from desensitization. B, Direct activation of nonfunctional mutants by 4BP-TQS. The same amount of cRNA was injected for each group, and recordings were performed after 3 days in 10–17 oocytes in each group). C, Co-application of 4BP-TQS with ACh or nicotine rescued more mutants. The same amount of cRNA was injected for each group, and recordings were performed after 3 days in 9–19 oocytes in each group. Asterisk (****) represents that the difference between the wild type and each mutant is statistically significant (P<0.0001) in Tukey multiple comparison test of one-way ANOVA. ♦♦, ♦♦♦, or ♦♦♦♦ represent the difference between blank and each mutant with statistical significance (P<0.01, P<0.001, or P<0.0001).

**Fig 6:**
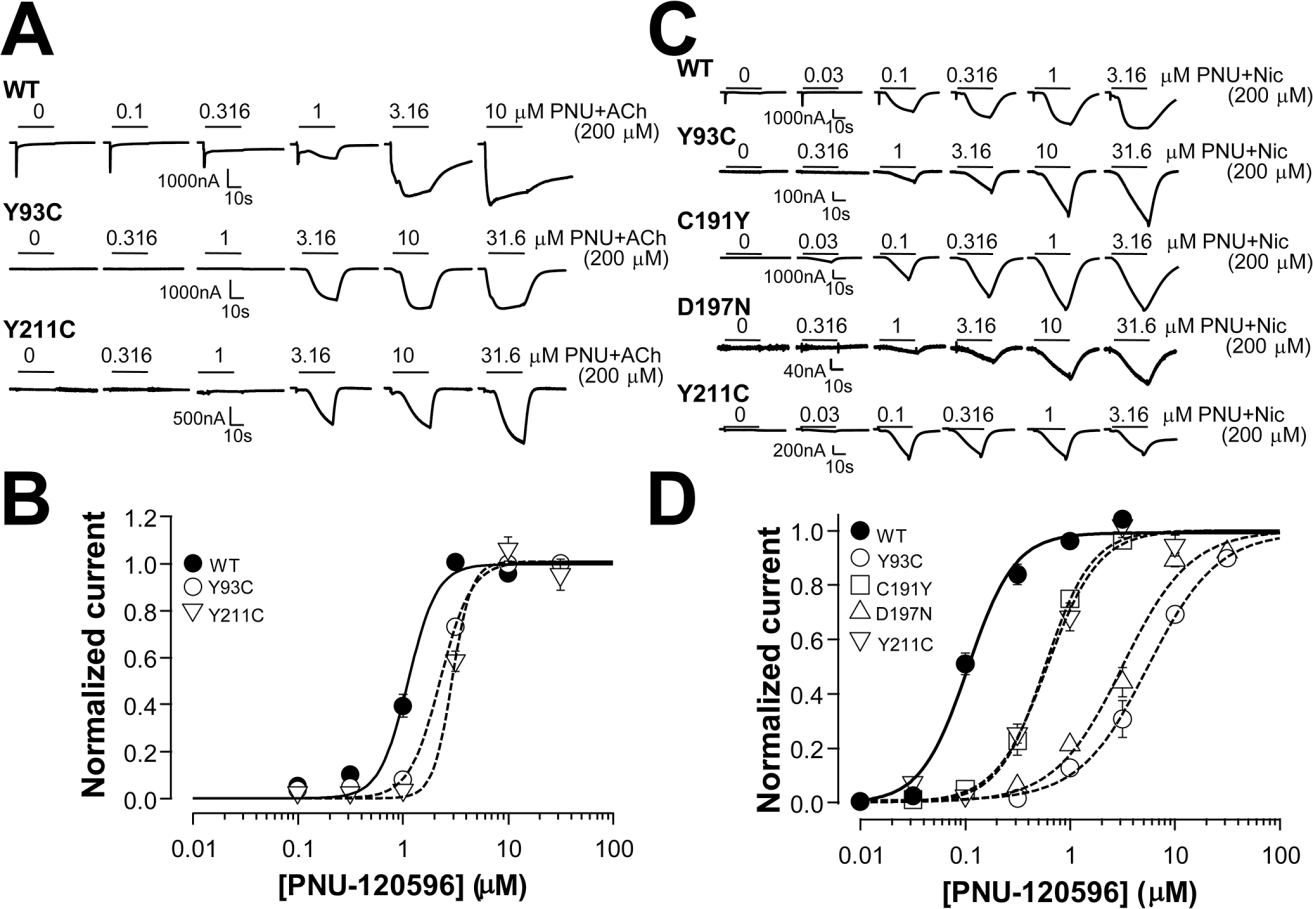
PNU120596 concentration-response for the rescued nonfunctional mutants with a fixed ACh or nicotine concentration. **A**. Representative current traces induced by increasing concentration of PNU-120596 in the presence of 200 μM ACh. **B**. Normalized and averaged (each group had at least 6 oocytes) current responses to ACh from A. Lines are nonlinear least squares fits of the normalized averages of the responses to the Hill equation. The derived EC_50_ values from individual fits are listed in Table 2. **C**. Representative current traces induced by increasing concentration of PNU-120596 in the presence of 200 μM nicotine. **D**. Normalized and averaged current responses (each group had at least 6 oocytes) to ACh from C. The derived EC_50_ values from individual fits are listed in Table 2.

The positive allosteric modulators (PAM) of α7 nAChR bind to the allosteric site in N-terminal domain or transmembrane domain to facilitate channel opening [31–33]. They have promising therapeutic potential [34]. The PAMs presumably reduce the energy barrier for channel opening. In fact, one PAM, PNU-120596, has been used to reveal function of some α7 nAChR silent agonists [35, 36]. Thus, it is possible that co-application of this PAM can rescue the nonfunctional mutant receptor. To test this hypothesis, we co-applied PNU-120596 with ACh or nicotine to all six non-responsive mutants. Indeed, some of the nonresponsive mutants became functional in the presence of PNU-120596. Fig 5A shows that PNU-120596 rescued ACh response in Y93C and R211C mutants, but not for the other 4 mutants. The rescue of the two mutants suggests that the mutants have surface expression, but their function is impaired due to mutation in the binding site in case of Y93C or the mutation in the coupling region in case of R211C. Interestingly, PNU-120596 rescued one more mutant (C191Y) in addition to Y93C and R211C for nicotine response. The nicotine response of another loop C mutant, D197N, could also be partially rescued. Although it was not significantly different from the blank control in initial ANOVA test, if we remove those groups with larger means (probably contributing larger variations to mask the groups with smaller values) in the ANOVA test, then it was significantly different from the blank control (P<0.0001). It was further confirmed by the concentration response (see Fig 6). However, the function of E173K and R206C mutants for either ACh or nicotine could not be rescued by PNU-120596.

**Table 2:** EC_50_ values for PNU-120596 with the fixed concentration of ACh/nicotine for the rescued mutants.

Mutant name	EC_50_ (μM) with ACh	EC_50_ (μM) with nicotine
WT	1.15±0.09	0.11±0.01
Y93C	2.09±0.10	5.22±0.62
C191Y	ND	0.60±0.05
D197N	ND	3.22±0.37
Y211C	2.91±0.20	0.67±0.10

ND: not detectable

**Fig 7:**
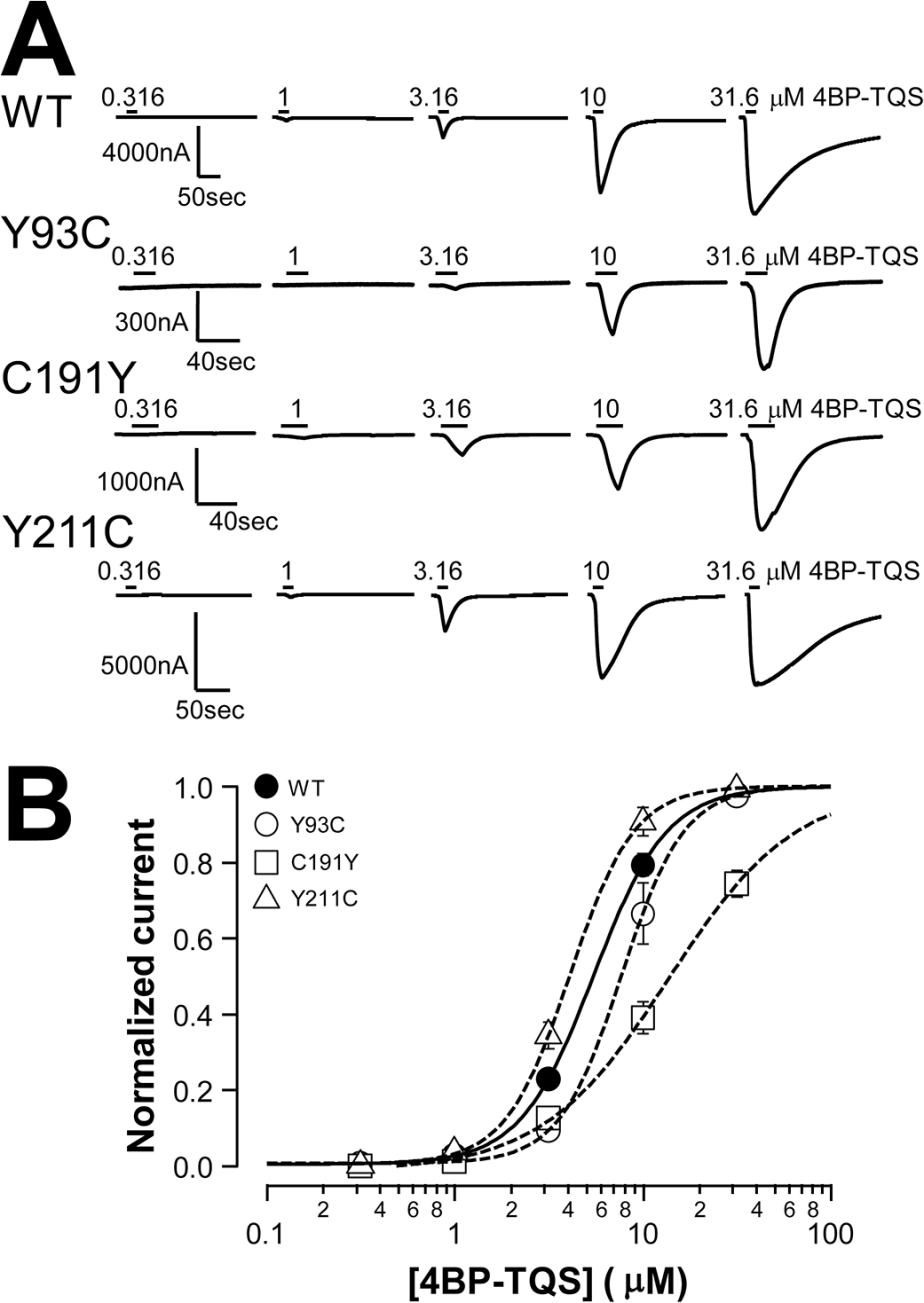
Concentration response of 4BP-TQS direct activation of the wild type control and Y93C, C191Y, and Y211C mutants. A, Representative current traces induced by increasing concentration of 4BP-TQS. B. Normalized and averaged current responses to 4BP-TQS from A. Lines are nonlinear least squares fits of the normalized averages of the responses to the Hill equation. The resulting EC_50_ values were 5.54±0.31, 8.21±1.03, 13.73±1.73, and 4.23±0.30 μM for the wild type control and Y93C, C191Y, and Y211C mutants respectively (n = 6 for each group).

Since the nonfunctional mutants have mutations in the orthosteric binding site or the coupling region between N-terminal domain and transmembrane domain, it is likely that these mutations do not influence the activation by an allosteric agonist. 4BP-TQS is a structural analog of an a7nAChR PAM, TQS. In addition to the PAM effect, it can also directly activate α7nAChR by binding to an allosteric site located in the second transmembrane, channel-lining, domain [37]. Fig 5B shows that 4BP-TQS directly activated 2 binding site mutants Y93C and C191Y, and 1 mutant in M1 (Y211C). Although the difference between Y93C and blank control did not reach statistical significance with ANOVA, it had the trend. The concentration response of 4BP-TQS for this mutant further supports that it could be activated by 4BP-TQS (see Fig 7). However, 4BP-TQS alone failed to activate E173K and D197N mutants, although both mutations are located in the N-terminal domain.

**Fig 8:**
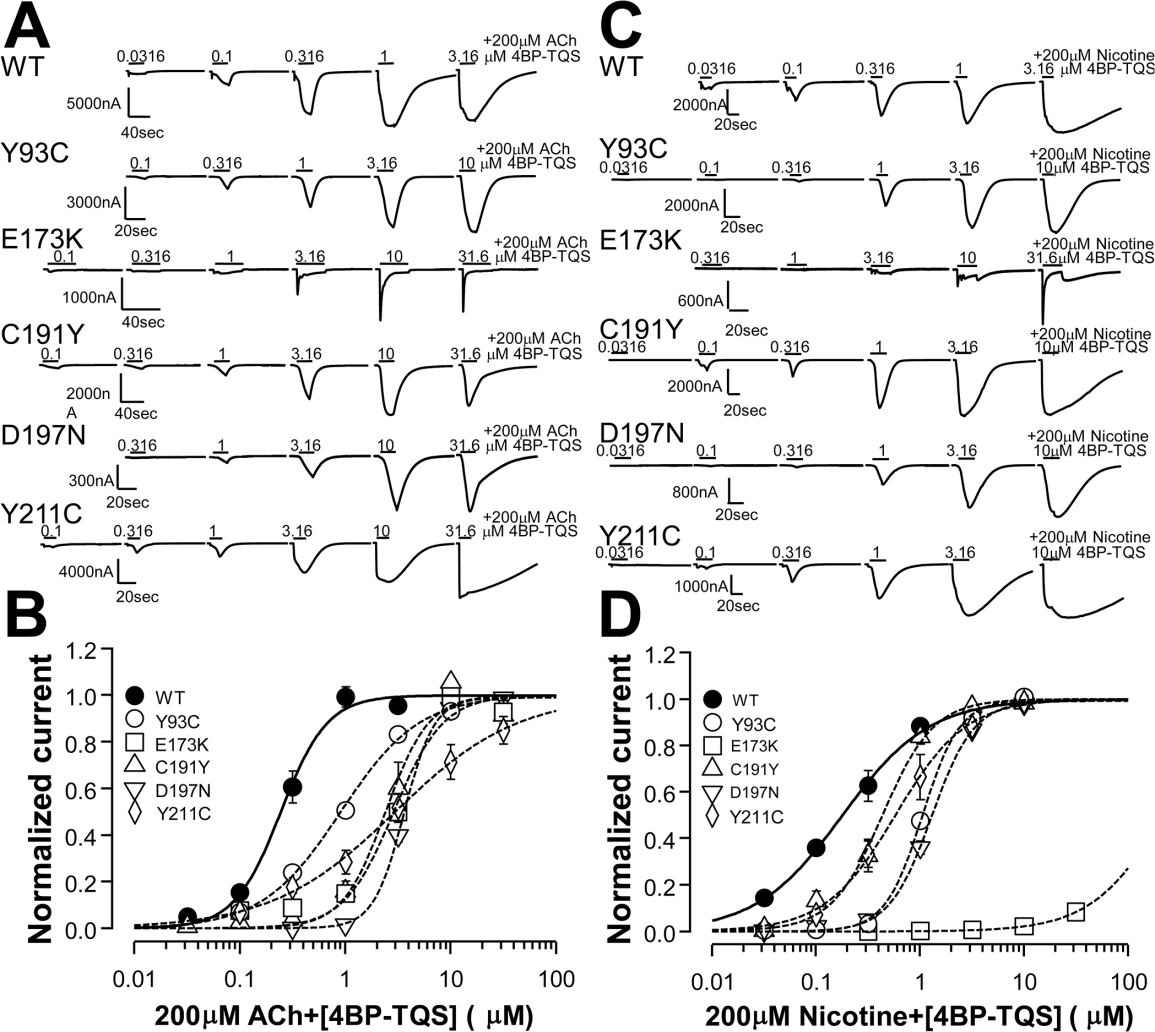
4BP-TQS concentration-response for the rescued nonfunctional mutants with a fixed ACh or nicotine concentration. **A**. Representative current traces induced by increasing concentration of 4BP-TQS in the presence of 200 μM ACh. **B**. Normalized and averaged (each group had 5–6 oocytes) current responses from A. Lines are nonlinear least squares fits of the normalized averages of the responses to the Hill equation. The derived EC_50_ values from individual fits are listed in Table 3. **C**. Representative current traces induced by increasing concentration of 4BP-TQS in the presence of 200 μM nicotine. **D**. Normalized and averaged current responses (each group had 5–6 oocytes) from C. The derived EC_50_ values from individual fits are listed in Table 3.

Since 4BP-TQS is also a PAM, it is also possible that it can rescue more nonfunctional mutants in the presence of an orthosteric agonist. Fig 5C shows that in the presence of ACh or nicotine, 4BP-TQS rescued more mutants than PNU-120596. Note that 4BP-TQS also showed a trend to rescue the ACh and nicotine effects on E173K and D197N mutant. Although the rescued currents did not reach statistical significance in initial ANOVA test, if we remove the groups with larger means (probably contributing larger variations to mask the groups with smaller values) in the ANOVA test, then the rescued currents in these two mutants for both ACh and nicotine were significantly higher than the blank control (P<0.0001). The rescuing effects in these mutants were further confirmed by their concentration responses of these mutants (Fig 8). However, the response to ACh or nicotine in the presence of 4BP-TQS for R206C mutant was essentially the same as the blank control.

**Table 3:** EC_50_ values for 4BP-TQS with the fixed concentration of ACh/nicotine for the rescued mutants.

Mutant name	EC_50_ (μM) with ACh	EC_50_ (μM) with nicotine
WT	0.27±0.04	0.19±0.03
Y93C	0.92±0.08	1.07±0.05
E173K	3.11±0.19	>31.6
C191Y	2.57±0.50	0.44±0.06
D197N	3.85±0.34	1.29±0.05
Y211C	5.50±2.74	0.70±0.14

### Concentration-response of the rescuing effect for nonfunctional mutants

To address whether the rescuing effect is concentration-dependent, we performed experiments with a fixed ACh or nicotine concentration in the absence and presence of increasing concentration of PNU-120596. Fig 6A and 6B show that with the fixed ACh concentration at 200 μM, PNU-120596 rescued the mutant receptor function concentration-dependently. Compared to the wild type receptor (rescued current from desensitization), 1.8 or 2.5-fold higher PNU-120596 concentrations were needed to rescue two mutant receptors. PNU-120596 rescued nicotine response in two more mutants C191Y and D197N, in addition to Y93C and R211C (Fig 6C and 6D). However, since the wild type PNU-120596 sensitivity is dramatically shifted to the left in the presence of a constant concentration of nicotine, the mutants showed larger difference (6-, 6-, 31-, 50-fold less sensitive than the wild type for mutants C191Y, R211C, D197N, and Y93C, respectively) from the wild type (rescued desensitization).

The concentration responses of 4BP-TQS direct activation of Y93C, C191Y, and Y211C are shown in Fig 7. Despite of non-responsiveness to ACh, the sensitivity of these three mutants to 4BP-TQS were not dramatically deviated from the wild type (1.5-, 2.5-, or 0.8-fold difference when compared to the wild type).

The rescuing effects of 4BP-TQS in the presence of an orthosteric agonist for five nonfunctional mutants were also concentration dependent. Fig 8A and 8B show the rescuing effect of 4BP-TQS for ACh responses of these mutants. Compared to the wild type receptor, 3-, 12-, 10-, 14-, or 21-fold higher 4BP-TQS concentration are required to rescue Y93C, E173K, C191Y, D197N, and Y211C mutants respectively, Note that unlike other mutants, the rescued current in E173K exhibited a relatively rapid desensitization, although the shift of EC_50_ for 4BP-TQS was not the highest among these five mutants. 4BP-TQS also rescued nicotine responses of these five mutants as shown in Fig 8C and 8D. While Y93C, C191Y, D197N and Y211C only showed moderate decrease (6-, 2-, 7-, 4-fold respectively) in sensitivity to 4BP-TQS in the presence of nicotine, E173K required greater than 163-fold higher concentration of 4BP-TQS to rescue its function. This is in contrast to a 12-fold increase in EC_50_ for 4BP-TQS with ACh. Similar to the rescuing effect with ACh, the rescued current of this mutant also exhibited rapid desensitization.

### Coexpression of nonfunctional mutants with the wild type

**Fig 9:**
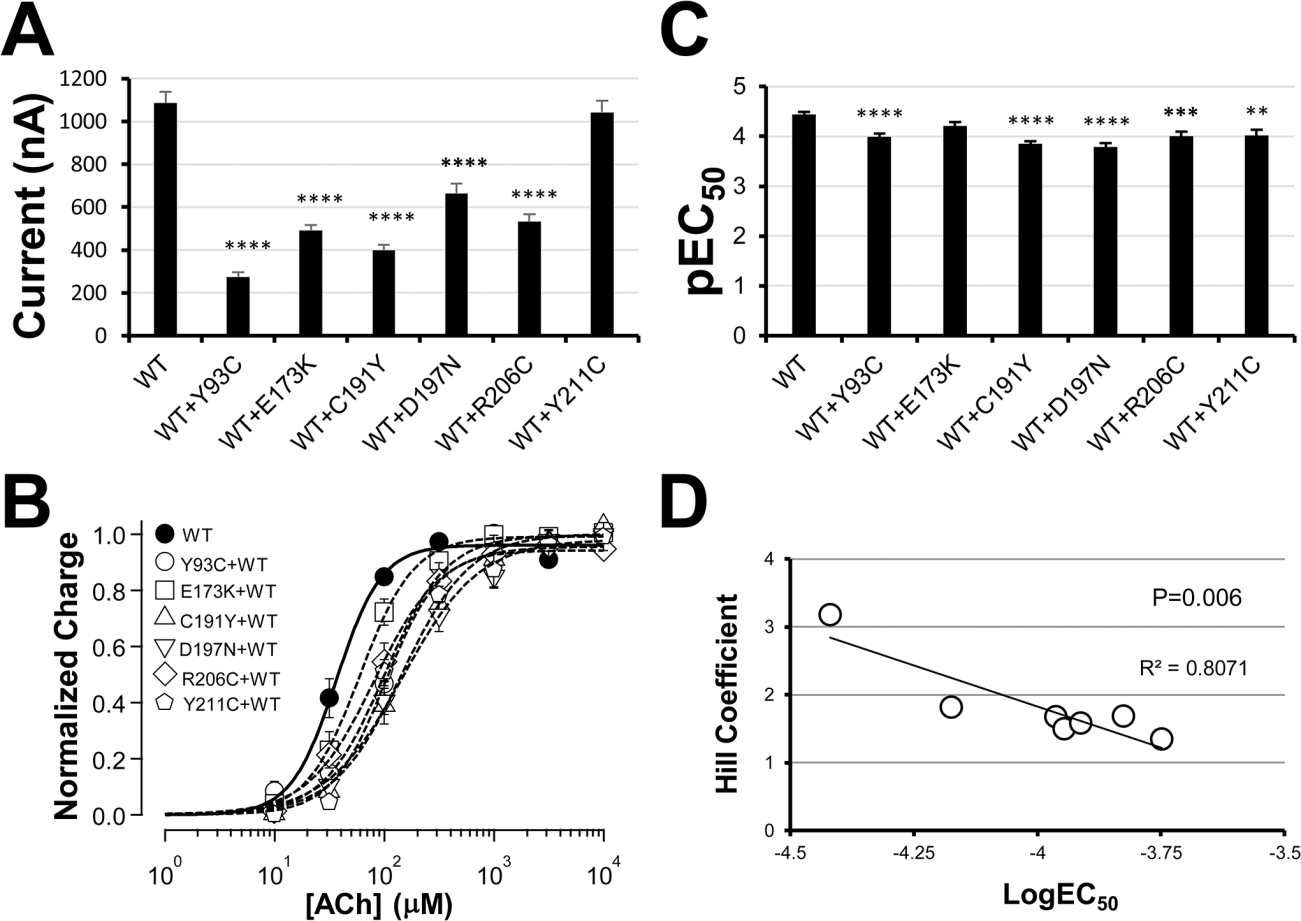
Coexpression of wild type and nonfunctional mutant. The same amount of cRNAs encoding the wild type or wild type plus mutant (in 1:1 ratio) were injected into *Xenopus* oocytes, and recorded with two electrode voltage clamp for ACh-induced current after 3 days of injection. **A**. Peak current induced by 3.16mM ACh for all groups on the 3 post-injection day. "****": P<0.0001. Each group had at least 16 oocytes. **B**. Averaged concentration response relationships of the wild type and wild type plus mutant as indicated. Each group is the average of six oocyte data. **C**. EC_50_ values were derived by fitting individual concentration-response curves in B. pEC_50_ values (negative logEC_50_) are used to plot the data. ("**", “***”, “****”: p<0.01, P<0.001, or P<0.0001). **D**. Linear regression analysis for the relationship between Hill slope and LogEC_50_.

Up to this point, our testing has been restricted to independent mutant receptors, which is equivalent to the homozygote condition. However, in real life, the frequency of heterozygote carriers is much higher than that of homozygote individuals. Thus, it is equally important to test whether heterozygote mimicking condition (coexpression of mutant and wild type) has impact on the receptor function. Fig 9A shows the currents induced by 3.16mM ACh for the 6 nonfunctional mutants separately coexpressed with the wild type. Coexpression of each mutant with the wild type was clearly functional. However, except for Y211C coexpression, all the other nonfunctional mutant coexpressions resulted in reduced ACh-induced current. The concentration-response relationships of the wild type and coexpressed mutants are plotted in Fig 9B. It seems that all mutant coexpressions could slightly reduce receptor sensitivity, since all mutant coexpressions show small rightward shift in their concentration-response relationships. By fitting the data to the Hill equation, we derived EC_50_ values for these concentration-response relationships. Fig 9C is the bar graph for pEC_50_ (negative log). Except for E173K coexpression, all the other mutant coexpressions exhibited slightly shifted EC_50_ values from the wild type receptor alone with statistical significance. Interestingly, we have also noticed that coexpression of the mutants with the wild type also reduced Hill coefficients to different extents. The mutant coexpressions with lower Hill coefficients tended to have larger EC_50_ shifts (Fig 9D). We will discuss this linear relationship later.

## Discussion

There are 55 SNPs causing missense mutations in the coding region of the human α7 nAChR gene in the NCBI SNP database. In this study, we selected and characterized 14 SNPs causing missense mutations in the agonist binding region and the coupling region between the amino-terminal domain and the channel gate in the transmembrane domain. In the oocyte expression system, we demonstrate that 6 out of 14 mutations made the receptors unresponsive to ACh and or nicotine in this expression system. Among remaining 8 mutants, 4 of them had reduced current expression, and one had a dramatic increase in ACh response but a dramatic decrease in nicotine response. Interestingly, some nonfunctional mutations could be rescued by α7 nAChR PAM, PNU-120596 or agonist-PAM, 4BP-TQS. Finally, when nonfunctional mutants coexpressed with the wild type, they could modify the receptor function in expression level or agonist sensitivity, suggesting a potential impact of these 6 SNPs on synaptic transmission even in heterozygous condition.

### Impact of the mutations on current level

Among 14 mutants tested, we found 4 of them showed reduced maximal whole-cell current expression and 6 of them were totally nonfunctional in the *Xenopus* oocyte system. Interestingly, W55G mutation dramatically increased ACh-induced current. Thus, except for two mutants (G212S and G212V) in the extracellular end of M1 domain and one conserved mutant in binding loop C, K192R, all the other mutants in the binding domain and coupling region exhibited different extent of alteration in whole-cell current level. Interestingly, while most of the mutants exhibited similar changes in ACh and nicotine responses, the W55G mutant exhibited an opposite change in ACh and nicotine response. Alteration of whole cell current could be due to a change in the number of channels expressed in the plasma membrane. Alternatively, it may result from the altered channel opening probability (gating efficiency) or changes in single channel conductance. In addition, it is also possible that the change in current level could be due to alteration in receptor channel kinetics, such as desensitization.

**Fig 10:**
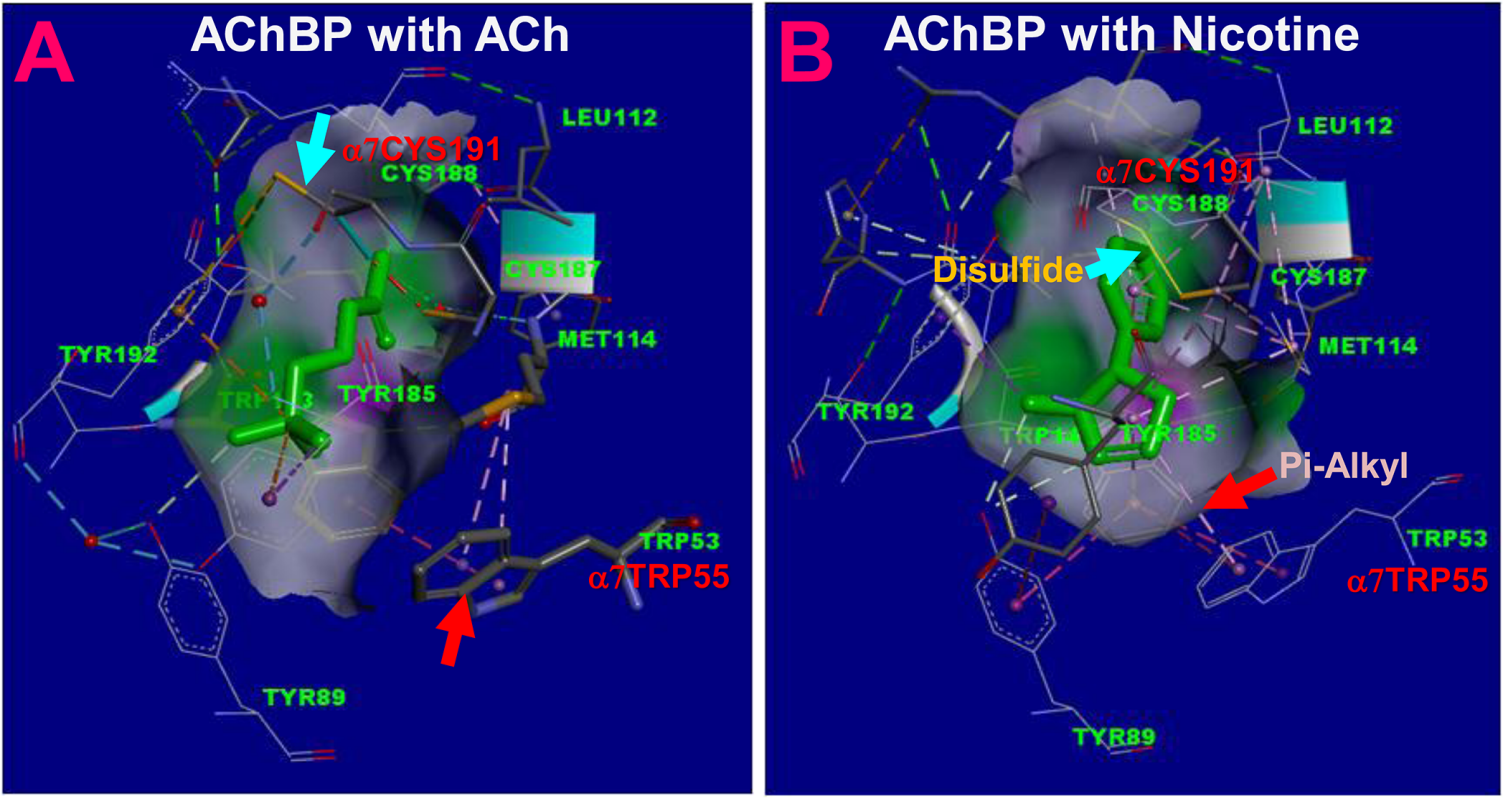
Different interactions of ACh and nicotine with the AChBP binding residues. A. The binding pocket between chain A and chain B of the AChBP co-crystalized with ACh (PBDID: 3WIP); B. The binding pocket between chain A and chain B of the AChBP co-crystalized with nicotine (PBDID: 1UW6). The residues of Cys191 and Trp55 in the human α7nAChR are labeled next to their homologous residues in the AChBP. Arrows indicate different interactions.

For nonfunctional mutants, the question is whether they are expressed on the cell surface. The function of the Y93C, E173C, C191Y, D197N, and Y211C could be rescued in the presence of a PAM for α7 nAChR, suggesting that they are expressed on the cell surface, but their function is disrupted by the mutations. Y93C and C191Y are the mutations in the binding site located in binding loop A and loop C. These two mutations most likely decrease the binding energy to such an extent that the channel cannot be opened upon agonist binding. However, in the presence of the positive allosteric modulator PNU-120596, the energy barrier for channel opening is reduced. Thus, the agonist can reopen the channel despite of the reduced binding energy for these mutant channels. Tyr93 is essential for orthosteric activation, and the mutation of this residue to cysteine makes the receptor insensitive to ACh, but still can be activated by an allosteric agonist [38]. Our results with 4BP-TQS direct activation of Y93C are consistent with that finding. Interestingly, for the C191Y mutant, rescuing effects of the PAM with ACh or nicotine are different. PNU-120596 could only rescue the nicotine effect but not the ACh effect. This phenomenon suggests that the binding energy loss for ACh is much higher than that for nicotine in this mutant. In the crystal structures of ACh binding protein (AChBP), a soluble protein homologous to the amino-terminal domain of nicotinic receptor, the homologous cysteine (CYS188) does not form the disulfide bridge with the neighboring cysteine (CYS187) when ACh bound to the receptor, but this cysteine residue can coordinate with another binding residue (TYR192) in the same loop C to interact with ACh (Fig 10A, [39]). Note that in the same pentameric structure of AChBP (3WIP chains A-E), chains A, D, E do not have a disulfide bond, but chains B and C do have a disulfide bond. This suggests that the receptor can adopt two different conformations when binding ACh. In contrast, when nicotine is in the binding site, the homologous cysteine can form a disulfide bridge with the neighboring cysteine in all five subunits of the pentameric structure and indirectly interacts with nicotine (Fig 10B, [40]). For the Y211C mutation, it is likely that the mutation decreases the coupling between the M1 and M2-M3 domains, the outward tilting of the latter is proposed to be the mechanism for channel activation for this receptor family [41]. In fact, a computational study predict that the activation pathway of this receptor family is via pre-M1 region [42]. In our homology model, this residue is facing the beginning of M3 domain and is likely to be interacting with Met261. The mutation of Y211C probably disrupts the coupling between pre-M1 to M2-M3 domain, making the receptor nonfunctional. However, weakening of gating energy by PN-120596 would allowed the weakened coupling to transduce the binding energy enough to open the channel. For D197N nonfunctional mutation, the nicotine but not the ACh effect could be partially rescued by PNU-120596. However, 4BP-TQS could partially rescue ACh and nicotine effect. This differential effect again suggests that nicotine and acetylcholine gate the channel differently, and PNU-120596 and 4BP-TQS modulate the channel differently. Asp197 is likely a key residue in coordinating the binding loops B and C. In fact, it forms a functionally important triad with Tyr188 and Lys145 (loop B) [43]. The homologous residue (Asp194) along with Lys139 (loop B) and Tyr185 in the α1 muscle type nicotinic receptor has been shown to be functionally important triad. Dynamic interaction of this triad is an important mechanism for channel activation [44]. For nonfunctional E173K mutant, Glu173 is located in loop 9. It has been reported that the mutation of this residue (E173A) could completely abolish the current, but preserve the surface expression of the mutant receptor [45]. Other mutations of this residue have similar effects, suggesting that Glu173 is an important coupling residue. Mutation of this residue can completely uncouple the agonist binding to the channel gating.

An alternative explanation of the PAM effect is that it makes the desensitized state conducting as proposed by Williams et al [46]. With this mechanism, the rescuable nonfunctional mutants can directly go to desensitized state upon agonist binding. However, in the presence of a PAMII, the desensitized channel is converted to a conducting state with different gating kinetics and even with different single channel conductance. Thus, they lack normal activation state, but still can be desensitized and converted to a conducting desensitized state by a PAMII. Regardless the mechanism, functional rescue of nonfunctional channel would have potential clinical applications.

4BP-TQS is an allosteric agonist as well as a PAM for α7nAChR. For the nonfunctional mutations in the orthosteric binding site or coupling region, if their surface expression is preserved, we expect that their response to 4BP-TQS direct activation through the allosteric site in the M2 domain should be normal. Indeed, 4BP-TQS directly activated Y93C, C191Y, and Y211C mutant receptors. The concentration response of 4BP-TQS for these mutants were similar to that for the wild type receptor, suggesting that mutations in the binding loop C tip, loop A, and M1 do not influence 4BP-TQS binding and function. However, in additional to sensitivity, we have noticed that the amplitude of the 4BP-TQS-induced currents in these 3 mutants was also different. With near saturation concentration, 4BP-TQS induced-current in Y211C had similar amplitude as the wild type. In contrast, Y93C and C191Y had the 4BP-TQS-induced currents with significantly lower amplitude, suggesting that these mutations may reduce the receptor surface expression. Alternatively, the mutations in the binding sites could allosterically influence channel gating efficiency. In GABA_A/C_ receptors, mutations of residues in the binding loops A, B, or E created spontaneously opening channels [47, 48]. Thus, it is possible that mutation of a residue in the binding site can have an impact on the channel gating. Perhaps Y93C and C191Y mutations not only influence binding, but also allosterically inhibit channel gating, resulting in lower gating efficiency by the allosteric agonist 4BP-TQS. In contrast, Y211C has no direct effect on channel gating as reflected by the full efficacy of 4BP-TQS when compared to the wild type. Its effect on the activation by orthosteric agonist likely due to uncoupling of the N-terminal orthosteric binding domain to the channel gating domain. This explanation is further supported by its similar 4BP-TQS sensitivity to that of the wild type. In comparison, the 4BP-TQS sensitivity of the Y93C and C191Y mutants were slightly reduced. For E173K and D197N mutants, unexpectedly, they could not be activated by 4BP-TQS alone, but their function could be restored with the collaborative effort of an allosteric agonist and an orthosteric agonist. Thus, the perturbation in the orthosteric binding loop C arm (D197N) or the coupling region (E173K) not only severely impair the coupling between the orthosteric binding site and the channel gate, but also completely abolish direct gating by allosteric agonist. The non-rescuable mutants could influence receptor assembly or might have a larger influence in binding or coupling energy. The conserved arginine at the middle position of the pre-M1 RRR motif of nicotinic receptor subunits is required for the transport of assembled α7 nAChR to the cell surface [49]. Thus, the corresponding R206C mutation would likely prevent surface expression of the receptor, most probably by disrupting interactions between pre-M1, loop2 and M2-M3 linker as suggested by molecular dynamics simulation in α7 nAChR [50] or experimental evidence in other subunits [51].

For the functional W55G mutant, our findings of its impact on ACh response are consistent with previous reports [30, 52]. In addition, our observation of differential impact of this mutant on ACh and nicotine efficacy is interesting. By examining the crystal structures of AChBP with ACh or nicotine, we found that nicotine can directly interact with TRP55 through a Pi-Alkyl interaction (Fig 10B). In contrast, ACh does not directly interact with TRP55 (Fig 10A). That could be an important mechanism for the differential impact of the mutation on the efficacy of ACh or nicotine

### Heterozygote mimicking expression for nonfunctional mutants

The frequencies of occurrence for the SNPs tested here are not determined, but they are likely to be relatively low. Thus, the homozygote individuals carrying these SNPs should be relatively rare. However, the heterozygous carriers of these mutations should be more frequent. To determine whether these nonfunctional mutants can influence the receptor function in heterozygote situation, we coexpressed the nonfunctional mutants (one at a time) with the wild type receptor. Our results (Fig 9) demonstrated that except for Y211C, other 5 nonfunctional mutant coexpressions with the wild type significantly decreased the maximal current when compared to the wild type subunit. The highest reduction of current was found in the Y93C coexpression, with 3.9-fold change. The fold change in remaining 4 groups were 2.2, 2.7, 1.6, and 2.0 for E173K, C191Y, D197N, and R206C. Thus, it is likely that the heterozygote individuals with these mutations have the reduced α7 nAChR function. Notably, despite only half of the amount of the wild type cRNA injected in Y211C mutant coexpression condition, the maximum current levels were not significantly different from the wild type. For R206C, since it is likely that the mutation can influence surface expression, the mutant subunit may not be able to co-assemble with the wild type subunit. Alternatively, it may get co-assembled with the wild type subunits with a relatively low efficiency. Incorporation of this mutant subunit into the receptor may also influence channel function as demonstrated in Fig 9A–9C. Y93C and C191Y are located in the binding site. Incorporation of these mutant subunit into the captor would reduce number of binding sites in a receptor. Although at single channel level, it has been demonstrated that one binding is enough to elicit full response in α7 nAChR[53], when the number of functional binding sites is reduced in a single receptor, the chance for the receptor to get a single binding for a given concentration of an agonist at any time would be reduced. This can clearly influence the receptor concentration response to an agonist. Thus, receptors with different number of functional binding sites would have slightly different sensitivities to an agonist. The mixed population of the receptors with slightly different agonist sensitivity can result in apparent shallow Hill slope.

Influence of E173K and D197N coexpression on the channel function likely through their influence in coupling between agonist binding to channel gating. For the ACh concentration-response relationship, all the receptors coexpressed with a nonfunctional mutant tend to be shifted to the right slightly and with a shallower Hill slope. Interestingly, the increase in EC_50_ (in log scale) is highly correlated to the decrease in Hill coefficient. This phenomenon suggests that co-assembly of the nonfunctional mutant subunit with wild type subunit (with different subunit stoichiometries) can result in functional channels with slightly reduced sensitivity. In this case, the difference in agonist sensitivity between all wild type and mutant containing receptors could be relatively small, so that different sensitivity components cannot be resolved, resulting in a shallow Hill slope when the data were fitted with the single Hill equation. However, the multiple sensitivity mixture can be recognized by the reduced Hill coefficient (especially when it is below unity).

Finally, although most of the SNPs we tested have been validated by multiple independent submissions to refSNP cluster, only 6 of them have been validated by the 1000 Genome Sequencing Project with the second generation of haplotype map (HapMapII). Another SNP has been validated by HapMap project. It should be kept in mind that except for the SNPs causing mutations of D47N and W55G, the remaining SNPs are located in a region (exons 5–10) that is nearly identical (with only 1 base difference) to that in CHRFAM7A gene with the partial duplication of the CHRNA7 gene. Thus, it is possible that some of the α7 SNPs could be the SNPs from CHRFAM7A gene, but mistakenly placed in the CHRNA7 SNPs. They need to be confirmed by haplotyping or other means in the future.

In summary, in this study, we have identified 11 α7 nAChR SNPs in the agonist binding and coupling regions that have a functional impact on the receptor. Among them, 4 SNPs were functional but with reduced current expression, 1 with increased ACh-induced current, but decreased nicotine-induced current expression, and 6 SNPs were nonfunctional. Among the functional SNPs, two exhibited slightly reduced sensitivity to ACh. Interestingly, 5 nonfunctional mutants can be rescued by the α7 nAChR positive allosteric modulator, PNU-120596 and/or 4BP-TQS. Nonfunctional mutants also influenced receptor function when coexpressed with the wild type in the heterozygote mimic condition. These changes of the receptor properties by the mutations would have potential impact on physiological function of the α7nAChR-mediated cholinergic synaptic transmission and anti-inflammatory effects. It would be interesting to see whether these abnormalities of receptor function can be correlated to cognitive functional differences or to an individual's anti-inflammatory ability in the future studies with genome wide association or knockin animal models. In addition, rescuing the nonfunctional mutants could provide a novel way to treat the related disorders.
